# Recent Advances and Perspectives of Nanomaterials in Agricultural Management and Associated Environmental Risk: A Review

**DOI:** 10.3390/nano13101604

**Published:** 2023-05-10

**Authors:** Sneha Tripathi, Shivani Mahra, Victoria J, Kavita Tiwari, Shweta Rana, Durgesh Kumar Tripathi, Shivesh Sharma, Shivendra Sahi

**Affiliations:** 1 Department of Biotechnology, Motilal Nehru National Institute of Technology Allahabad, Prayagraj 211004, India; 2 Department of Physical and Natural Sciences, FLAME University, Pune 412115, India; 3 Amity Institute of Organic Agriculture, Amity University Uttar Pradesh, Noida 201313, India; 4 Department of Biology, St. Joseph’s University, 600 S. 43rd St., Philadelphia, PA 19104, USA

**Keywords:** nanomaterials, agri-nanotechnology, nanoherbicides, nanofertilizers, nano-emulsions

## Abstract

The advancement in nanotechnology has enabled a significant expansion in agricultural production. Agri-nanotechnology is an emerging discipline where nanotechnological methods provide diverse nanomaterials (NMs) such as nanopesticides, nanoherbicides, nanofertilizers and different nanoforms of agrochemicals for agricultural management. Applications of nanofabricated products can potentially improve the shelf life, stability, bioavailability, safety and environmental sustainability of active ingredients for sustained release. Nanoscale modification of bulk or surface properties bears tremendous potential for effective enhancement of agricultural productivity. As NMs improve the tolerance mechanisms of the plants under stressful conditions, they are considered as effective and promising tools to overcome the constraints in sustainable agricultural production. For their exceptional qualities and usages, nano-enabled products are developed and enforced, along with agriculture, in diverse sectors. The rampant usage of NMs increases their release into the environment. Once incorporated into the environment, NMs may threaten the stability and function of biological systems. Nanotechnology is a newly emerging technology, so the evaluation of the associated environmental risk is pivotal. This review emphasizes the current approach to NMs synthesis, their application in agriculture, interaction with plant-soil microbes and environmental challenges to address future applications in maintaining a sustainable environment.

## 1. Introduction

Nanotechnology is a novel approach with potential to manipulate the physicochemical properties of substances at the molecular level for the development of innovative products. A boom in nanotechnology research has been experienced over recent years. It is impacting our everyday lives and leaving influential footprints in our society [1]. It has wide usage in all industrial sectors, be it pharmaceuticals, food, animal feeds, cosmetics, electronics or agricultural production [2,3,4].

Agriculture supports the economy of developing nations by providing food, fabric, wood and raw materials for several industries. The Food and Agricultural Organization (FAO), in 2019, estimated that global demand for agricultural production must increase by 25–70% by 2050 to address the food crisis in the growing human population. Several challenges such as climate change, rampant usage of chemical fertilizers, soil contaminants, exploitation and deterioration of soil and water resources plague agricultural productivity. Current advancements in nanotechnology research are showing substantial impact on agriculture development and precision farming. The application of nano-enabled products in agriculture maximizes agriculture output (i.e., yield) while minimizing agrochemical input (herbicides, pesticides and fertilizers) by administering controlled and targeted actions [2].

The implementation of nano-based tools and techniques can immensely improve agricultural products. The nanosized materials (1–100 nm in at least one dimension) have diverse physicochemical properties, high catalytic reactivity, solubility and biochemical activity, due to their high surface-to-volume ratio [5]. NMs used in crop improvement and sustainable agriculture can be of natural origin (i.e., naturally formed in the final product) or intentionally added. Intentionally added NMs can be synthesized by physical, chemical and biological methods [6,7,8]. Synthesized NMs can be categorized into organic NMs, metals, metal oxides and carbon-based nanostructures [9]. Each NM has its own set of characteristics and applications. NMs are potentially used as nanofertilizers, nanoherbicides and nanopesticides with targeted action and delivery of active components, as nanosensors to detect environmental changes and nutrient requirements and also as catalysts in soil and ground water remediation [10,11,12]. Application of zinc, titanium, iron, silicon and selenium nanoparticles (NPs) has been reported to induce a tolerance response against stress and improve crop productivity [13,14,15]. As the world is grappling with environmental degradation and global food security issues, qualitative and quantitative expansion of crop productivity is pivotal. It is believed that NMs can be better tools for sustainable agriculture.

NMs have well-known applications in various industries. Despite the potential benefit of nanotechnology in the agriculture sector, very few nano-enabled agro inputs have made their way to the market [16]. The primary causes of the lack of commercialized nano-agricultural products in the market are limited knowledge concerning nanomaterial biosafety, regulatory guidelines, adverse effects, fate and interaction with the biological system once they are disseminated into the environment. The potential risks of NMs are still inconclusive and are under active research. This review briefly covers recent advances from the agricultural nanotechnological aspect and provides insight into toxicological fundamentals and risk assessment of NMs for legislation, as well as public awareness and acceptance.

## 2. Methods of NMs Synthesis

There are three different approaches, physical, chemical and biological, adopted for the synthesis of NMs. The selection of synthesis method is crucial for the properties of NMs as these significantly control their size, surface coating and their interaction with living cells. The desired characteristics of NMs can be achieved by using suitable reducing agents and synthesis methods. The different techniques used in NMs synthesis are briefly described in this section.

Two basic approaches for manufacturing nanosized materials, a top-down approach and a bottom-up approach, are discussed as follows.

### 2.1. Top-Down Method

It is a destructive method where bulk materials are pressed or crushed down into the nanometer size range through a mechanical approach. This approach includes techniques such as laser ablation, electro-sputtering, ball milling, lithography, thermal evaporation and sputtering [17]. The top-down method generally includes physical methods.

#### Physical Methods

The physical method includes different approaches. Ball milling is a very effective method for synthesizing carbon NMs which provides solutions for environmental remediation, energy storage and conservation demands. Mechanical milling methods are generally used for the preparation of metal nanoalloys (nickel, aluminium, copper, etc.) and other nanocomposites. This method is very cost effective for producing very small (2–20 nm) NMs [17,18]. The electrospinning method is the simplest method for the production of micro- and nanofibers. Modified electrospinning includes coaxial electrospinning that can be used to form long, ultra-thin fibers. This is a simple and versatile method used for the manufacturing of core–shell, polymer (inorganic, organic and hybrid) and hybrid NMs [19]. Lithography is another valuable technique for the production of nano architectures through the use of a focused electron or light beam. The two most common types of lithographic techniques are masked and unmasked lithography. Soft, nanoimprint and photolithography are examples of masked lithography. The unmasked lithography method includes focused ion beam, electron beam and scanning probe lithography [20,21]. Sputtering is a unique process for creating thin films of NMs by bombarding the surface of the material with high-energy particles such as plasma or gaseous ions. Sputtering is very special because the composition of the sputtered material remains the same as the target material with fewer contaminations, and it is more economical than electron beam lithography. The advanced laser ablation technique utilizes a high-energy laser beam to vaporize precursor material. This method is considered a green technique for producing a wide range of high-purity NPs in the quantum size range (< 10 nm) [22].

### 2.2. Bottom-Up Approach

Bottom-up manufacturing involves the building up of the atom or molecules to form NPs. This technique encompasses chemical reactions to assemble the basic units (atoms or molecules with nuclei) at the nanoscale. The bottom-up approach is a better technique, resulting in good surface properties and particle size because of the self-assembly of the materials used.

#### 2.2.1. Chemical Method

The chemical reduction process is the most common chemical method for NMs synthesis. The chemical approach includes processes such as microemulsion, sol–gel methods, hydrothermal reduction, co-precipitation and thermal reduction. For the preparation of carbon-based NMs, chemical vapor deposition (CVD) methods are crucial. The ideal precursor for chemical vapor depositions must have adequate volatility and good stability, should be non-hazardous, cheap and chemically pure and have a good shelf life. In CVD, a thin film of NMs is formed via the deposition of the gaseous precursor at a very high temperature. It is an effective approach for the production of high-quality two-dimensional NMs [23]. The sol–gel method is a wet chemical approach that is extensively utilized for the manufacturing of NMs (MNMs). In this method, a liquid precursor is first transformed into the sol, which is later converted into a gel network. This method is very effective for manufacturing high-quality metal-based NMs. The method is more economical and has several advantages, such as the low processing temperature, homogeneity of the generated material and the ease with which it can be used to produce high-quality nanocomposite and nanostructures. The hydrothermal method is also widely employed for the manufacturing of a variety of NMs, such as nanorods, nanosheets, nanowires and nanospheres [24]. A hydrothermal method utilizes the aqueous phase at high pressure and at a critical temperature in a closed vessel. Hydrothermal methods combined with microwaves have gained significant attention due to the benefits yielded by microwaves. Solvothermal and hydrothermal processes are identical; the only difference being the occurrence of non-aqueous phase in solvothermal process [25]. The reverse micelle approach includes water in an oil emulsion system, where the water core acts as a nanoreactor for the synthesis of NPs. The water and oil ratio is the size-controlling factor in the reverse micelle approach. In this method, water concentration primarily affects the size of synthesized NPs; therefore, tiny water droplets form smaller NPs [26]. In comparison to physical methods, chemical methods have several benefits, such as high yield and low cost. However, the use of toxic and hazardous chemical-reducing agents can pose harm to the environment.

#### 2.2.2. Biological Methods 

**Figure 1:**
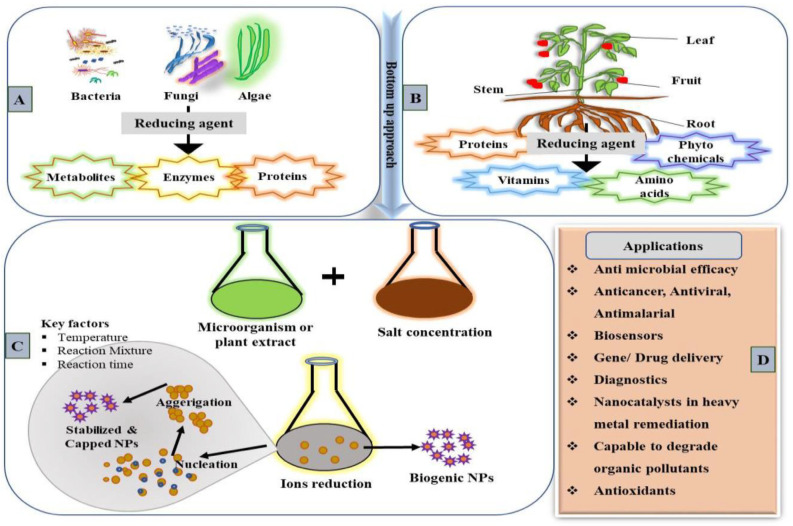
Systematic representation illustrating the various biological systems used for the biogenic synthesis of NMs. (**A**) Different microorganism sources for NP synthesis and their reducing factors [27]. (**B**) Plant parts and the phytochemicals used in NPs synthesis. (**C**) Catalytic activities and factors involved in biogenic synthesis. (**D**) Biogenically synthesized NPs’ diverse application in various fields.

Biological methods have emerged as a viable solution to overcome the limitations of traditional physical and chemical synthesis methods. Biological approaches are simpler, cost effective and eco-friendly. Biogenically synthesized NPs are easy to characterize and superior in quality to traditionally synthesized ones [27]. Even though physical and chemical methods can form a large quantity of NMs in a short time, they are cumbersome, expensive and release toxic chemicals detrimental to the biotic as well as abiotic components in the environment. In the last 10 years, a spurt in academic articles on the green synthesis of NPs has been observed every year. The biological synthesis of NPs can be achieved by using various biological systems, including plants, plant products, bacteria, fungi, yeast and viruses (Figure 1) [28,29,30].

##### Microbe-Mediated Synthesis

An account of their ubiquity in the environment, fast growth, easier cultivation and ability to adapt to ambient pH, temperature, pressure and environmental conditions showed that microorganisms are suitable machinery for the biosynthesis of NPs. The mechanism of NPs synthesis through biological agents varies from organism to organism [31,32,33]. Microbial synthesis can be extracellular or intracellular depending on the type of reaction condition [33,34]. Table 1 summarizes various NPs synthesized by microbes as biological agents. Fungal synthesis of NPs has been explored by exploiting bioactive compounds and metabolites from fungi. These microorganisms are an attractive agent for the synthesis of silver NPs due to their heavy metal tolerance, biomineralization and metal accumulation abilities. Several different fungal strains have been used to synthesize silver NPs, such as *Aspergillus flavus* [35], *Guignardia mangiferae* [36], *Aspergillus versicolor* [37], *Cladosporium cladosporioides* [38], *Beauveria bassiana* [39], *Penicillium oxalicum* [40], *Bjerkandera* sp. [41], *Aspergillus* sp. [42], *Aspergillus oryzae* [43] and *Aspergillus terreus* [44]. Intracellular synthesis involves exposure of metal precursors to a fungal mycelia culture that results in metal internalization and reduction inside the cell. After synthesis, additional treatment is required to release the NPs [44,45]. While extracellular synthesis is more economic, as NPs are synthesized using only cell-free fungal filtrate containing bioactive compounds, this method is more appropriate because no post-treatments are required to harvest NPs from the cell. Dispersed NPs can be easily purified using simple processes such as filtration, dialysis and gel filtration [46,47]. Many enzymes can initiate the process of NPs synthesis, but nicotinamide adenine dinucleotide (NADH) and NADH-dependent nitrate reductase enzymes are accounted for the microbial based synthesis of metal NPs. Recently, Hietzschold et al. reported that NADP can be solely responsible for the reduction of silver nitrate to form silver NPs [48].

Owing to the biosorption properties, intracellular sequestration, extracellular precipitation and efflux pump for metal tolerance, bacteria are the suitable candidates for metal ion reduction and NPs formation [7,49]. Researchers have successfully synthesized different NPs from the bacterial strains *Pseudomonas deceptionensis* [50], *Pseudoduganella eburnean* [51], *Bacillus subtilis* [52] and *Cuprividus* spp. [53].

Actinomycetes, the filamentous soil bacteria, are famed for producing bioactive compounds to survive in harsh environmental conditions. These bioactive compounds have received more attention due to their high stability, commercial value, unique antimicrobial properties and uncommon substrate specificity. The genus *Streptomyces* is known for its significant contribution to secondary metabolite production with high commercial value [54]. Many researchers explored this property of the *Streptomyces* genus and used biomass filtrate as a reducing agent for the green synthesis of silver NPs. The synthesized NPs were crystalline, spherical and had an average size of less than 100 nm with a surface plasmon resonance absorption band at 400–450 nm [55]. In another study, silver NPs were synthesized by using haloalkaliphilic *Streptomyces* spp. Ag NPs synthesized through *Streptomyces* spp. were spherical in shape with an average diameter of 16.4 ± 2.2 nm and had significant phytopathogenic activity against *Fusarium verticillioides* and *Ustilago maydis* [56]. Extracellular production of zinc and gold NPs through *Streptomyces* spp. was also reported [57,58]. The NPs produced by green synthesis were found to be more stable because of the presence of natural biomolecules that act as capping and stabilizing agents.

Several studies demonstrated that viruses can serve as a versatile platform for nanoscale product formation [59,60]. Viral biosynthesis provides a broad range of shapes, sizes, compositions and physicochemical properties of NPs. Material scientists use virus capsids as bio-templates for the development of novel nanohybrid materials. Plant viral capsid proteins are suitable biofactories for the fabrication of a wide range of NMs. They offer desired properties with inorganic and organic moieties integrated in a very precise and controlled manner [60]. The advantages of using viral proteins for NMs synthesis include ease of chemical and genetic manipulation, degradability, nontoxicity for humans and a well-known atomic structure with a possible ligand-attaching site [61,62]. In a study by Ahiwale et al., gold nanoparticles were prepared by using a rare bacteriophage of the podoviridae family. Viral-inspired Au NPs were found to be in the 20–100 nm size range. They showed antibiofilm activity against the human pathogen *Pseudomonas aeruginosa* at a very low concentration of 0.2 mM [63]. Recently, nanotechnologists exploited the plant virus squash leaf curl China virus (SLCCNV) for gold and silver NPs fabrication. A virus–metallic nanohybrid (Au and Ag) was synthesized using the pH-activated capsid of SLCCNV, and its electrical conductivity was also determined for biomedical applications [64]. Several studies illustrated the viral-mediated synthesis of different nanostructures such as platinum nanotube [65] and viral-like NPs (VLN) [66] and cadmium sulfide (CdS) nanocrystal [67], gold and iron oxide NPs [68]. A large number of viral-mediated NPs studies are available, but their application in agricultural practices or against plant pathogens is yet to be explored.

**Table 1:** Biological synthesis of MNPs using a diverse group of microbes.

Nanoparticle Synthesized	Source	Size	Application	Ref.
Ag NPs	Bacteria			
	*Bacillus endophyticus*	5 nm	Antimicrobial activity against *Candida albicans*, *Escherichia coli*, *Staphylococcus aureus*	[69]
	*Sinomonas mesophile*	4–50 nm	Antimicrobial activity against multi-drug-resistant *S. aureus*	[70]
	*Pantoea ananatis*	8.06–91.31 nm	Antimicrobial activity against multidrug-resistant bacteria and efficient against some pathogenic microbes as well	[71]
	*Pseudomonas strain*	20–70 nm	Showed highest antibacterial activity	[72]
	Fungi			
	*Aspergillus terreus*	16–57 nm	Efficacy in antibacterial activity	[73]
	*Penicillium aculeatum*	4–55 nm	Antimicrobial agent, drug delivery vehicle (anticancer drug)	[74]
	*Fusarium oxysporum*	5–13 nm	Antibacterial and antitumor activities	[75]
	*Metarhizium anisopliae*	28–38 nm	Antimalarial activity	[76]
	Algae			
	*Portieria hornemannii* (Red algae)	60–70 nm	Alternative to antibiotics which are commercially available against fish pathogens	[77]
	*Padina* sp. (marine algae)	~25–60 nm	Antibacterial and antioxidant activities	[78]
Au-Ag/ Ag NPs	Bacteria			
	*Stenotrophomonas*	Silver (40–60 nm) and Gold (10–50 nm)	-	[79]
	*Bacillus subtilis*	20–25 nm	Dye degradation	[80]
	*Mycobacterium* sp.	5–55 nm	Anticancerous activity	[81]
	Fungi			
	*Cladosporium cladosporioides*	60 nm	Antibacterial and antioxidant activities	[82]
	*Aspergillus* sp.	2.5–6.7 nm	Biocatalysis of nitrophenol compounds	[83]
	*Rhizopus oryzae*	16–43 nm	Hemocompatible activity	[84]
	Algae			
	*Gelidiella acerosa* (Marine algae)	58–117.6 nm	Antidiabetic, antibacterial and antioxidant activity	[85]
	*Cystoseira baccata* (Brown algae)	8.4 nm	Cancer therapies	[86]
	*Pithophora oedogonia*	32.06 nm	Electrocatalytic activity by determining the presence of carbendazim molecules in soil	[87]
Cu NPs	Bacteria			
	*Shewanella loihica*	10–16 nm	Antimicrobial activity	[88]
	*Shewanella oneidensiS*	20–40 nm	Biocatalysts	[89]
Se NP_S_	Bacteria			
	*Lysinibacillus* sp.	100–200 nm	Photocatalytic activity	[90]
	*Bacillus subtilis*	50–400 nm	H_2_O_2_ sensoristic device	[91]
CdS NPs	Bacteria			
	*Escherichia coli*	2–5 nm	–	[92]
	*Pseudomonas aeruginos*	20–40 nm	Removal of heavy metasl as cadmium	[93]
TiO_2_ NPs	Bacteria			
	*Bacillus mycoides*	40–60 nm	Qantum dot sensitized solar cells	[94]
	*Aeromonas hydrophila*	28–54 nm	Antibacterial activity	[95]
ZnO NPs	Bacteria			
	*Bacillus licheniformis*	40–400 nm	Photocatalytic activity, dye degradation and bioremediation	[96]
	*Serratia nematodiphila*	10–50 nm	Antimicrobial as well as antifungal activity	[97]
	Fungi			
	*Candida albicans*	25 nm	Synthesis of steroidal pyrazolines	[98]
	*Aspergillus terreus*	10–45 nm	Antibacterial, cytotoxic activity, UV protection	[99]
	Algae			
	*Chlorella extract* (Microalgae)	20 nm	Showed photocatalytic activity	[100]
	*Sargassum muticum*	30–57 nm	Beneficial cytotoxic effect on human liver cancer cells	[101]

##### Plant-Mediated Synthesis

The green synthesis approach for NMs synthesis aims to develop a method, reagent and process that substitute remediation or decreases the release of toxic chemicals to ensure the safety of the environment. Plants are the richest source of a diverse group of chemicals, such as terpenoids, proteins, amino acids, tannins, phenols, flavones, saponins, alkaloids and polysaccharides, that is actively involved in the reduction of metals. Because of the presence of these phytochemicals, the harnessing of plant materials for NPs synthesis has been appraised as more reliable as well as eco-friendly [102,103,104]. Different plant parts, including stem [105], root [106], seeds [107], leaves [108], fruits [109], bark [110] and flowers [111], have been explored for NPs synthesis (Table 2).

## 3. Nanomaterials for Agricultural Application

Nanotechnology offers a multifold scope for agricultural advancements by usage of NMs. Several fields such as biomedicine, food, energy, defense, textiles, paints and home goods have examined the use of and benefits presented by nanotechnology on a regular basis. Techniques such as nano-priming for rapid seed growth and increasing crop yield through the use of nanofertilizers, nanopesticides and nanoweedicides, etc., are proving to be a panacea for agriculture.

### 3.1. Nanomaterials in Crop Production

Nanotechnology as a broad area of research has come up with numerous applications in agronomy that have benefited agriculture through the improved yield of crops and the accelerated germination process of seeds and plant growth as well. The biological process of seed germination is complex, and it depends on the soil’s properties, genetic makeup and environmental conditions. Recent studies have demonstrated that NPs, including carbon nanotubes (CNTs), silicon dioxide (SiO_2_), zinc oxide (ZnO), titanium dioxide (TiO_2_) and gold (Au) NPs, have eased the germination of seed in crops such as wheat, pearl millet, tomato soybean, barley, rice and maize [122,123]. TiO_2_ NPs have been found to enhance seed germination by significantly decreasing the mean time for germination in *Agropyron desertorum* [124]. Furthermore, non-metallic NPs such as multiwalled carbon nanotubes (MWCNTs) can promote the germination of seeds in a variety of crops by improving the seed’s capacity to absorb water [125]. Additionally, NPs increase the tolerance of plants against abiotic stress primarily by scavenging reactive oxygen species (ROS) and increasing enzyme antioxidant activity [126]. Graphene NPs increase alfalfa’s resistance to alkaline circumstances, specifically by enhancing the activity of antioxidant enzymes and boosting seedling root length along with its fresh and dry weight [127].

Nano-priming is a seed priming method that contributes to increased seed germination, growth and yield. Many studies claimed a potential increase in the germination of seeds and seedling efficiency in crops such as wheat and tomato [127,128,129]. Yang et al. showed that phytohormones are responsive to NP treatment [130], while the content of indole acetic acid (IAA) and abscisic acid (AB) has been reported to increase in the roots of transgenic and non-transgenic rice in response to Fe_2_O_3_ [131]. Nano-priming is considerably effective under stress conditions, where it enhances the percentage of seed germination, length of root and shoots and seed vigor index [132]. *Calendula officinalis* seeds primed with silicon NPs exhibited improved antioxidant activity and total flavonoid content under drought stress. Thus, under the stress condition, priming with nanosilicon improves *C. officinalis*’s physiological and metabolic characteristics [133]. Another major role of NMs in crop production is the controlled delivery of materials (pesticides and fertilizers) via nanoencapsulation, which presents a meticulous option for crop enhancement while maintaining the surrounding environmental health. Formation of nano-agrochemicals that can regulate the nutrient release, and, as well, maintain the health and fertility of the soil by providing it with the beneficiary elements, is being studied. While external elements such as rain and wind can easily leach fertilizers from the application site, porous NPs encapsulate the fertilizer and retain it in the soil. This characteristic extends the fertilizer release period and enhances both the chemical and physical characteristics of the soil [134]. For reducing chemical pesticide doses, boosting crop productivity and fostering sustainable development, nanoformulation or nanoencapsulation of insecticides, herbicides, fungicides and bactericides with NMs hold immense potential.

### 3.2. Application of Nanomaterials as Herbicides, Pesticides and Nanofertilizers

In recent years, nanotechnology with smart nanoscale carriers for effective delivery of macro- and micronutrients, plant growth regulators, pesticides and fertilizers has become an efficient method for sustainable agriculture [135,136]. Nano-carriers prevent chemical discharge and solve environmental issues by securing plant roots to the organic materials and soil structure in the ecosystem. These aids in enhancing the bioavailability of active ingredients to the plant while lowering the effort and waste product [137]. Research on herbicides clearly places a higher priority on lowering the non-target toxicity of the herbicides through the use of NPs. De Oliveira et al. showed that pre-emergence application of solid lipid nanoparticles containing atrazine and simazine was more effective at eliminating the target plant *Raphanus raphanistrum* than post-emergence use of the herbicide alone [138]. Chidambaram et al. (2016) examined the loaded nano-sized rice husk waste particles with 2,4-dichlorophenoxyacetic acid (2,4-D). They discovered that NPs loaded with 2,4-D herbicide had superior herbicidal action to 2,4-D alone against the target plant (*Brassica* sp.). It was also evaluated that herbicides loading at rice husk reduced the leaching effect in soil [139]. In comparison to paraquat alone, Dos Santos Silva et al. employed paraquat loaded with alginate or chitosan, which reduced the leaching of herbicide in soil sorption experiments [140].

Similar to nanoherbicides, nanopesticides are also garnering recognition for replacing traditional pesticides due to their enhanced efficacy against a variety of pests and potential for tailored action, which lowers the environmental toxicity. In a study with *Drosophila melanogaster* as a model organism, the anti-pest activity of chitosan loaded with permethrin and spinosad was tested at various doses. It demonstrated that the combination of spinosad and permethrin in chitosan is more effective than either compound alone, suggesting that nanoformulations may be used for pest control management [141]. Further, the association of chitosan with zinc helps enhance the plant’s immunity. According to Choudhary et al., the formulation of zinc and chitosan nanoparticles increases the antioxidants and content of lignin in maize crops to elevate disease control [142]. Red-seaweed-extract-derived silver nanoparticles are suited for use in the formulation of nanopesticides due to their antibacterial and antifungal properties [143].

As crop plants can only absorb 30–50% of chemical fertilizers, a sizable portion of the input remains in the soil, which causes soil sterility and ground water contamination. Due to saturation, fertilizer efficiency thus declines over time [144]. Nanofertilizers have efficiency to reduce the nutrient loss via controlled release and thus may minimize the amount of fertilizer application in the field [145]. According to the available literature, nanofertilizers are benefiting several crop yields [146,147,148,149]. Gatahi et al. (2015) studied the impacts of nanobiofertilizer in tomato crops affected by *Ralstonia solanacearum*-caused bacterial wilt disease and its pest-resistant function against wilt disease [150]. Gouda et al. examined the protective effects of nanobiofertilizers that contain PGPR (*Pseudomonas* sp., *Bacillus subtilis* and *Paenibacillus elgii*) on leguminous crops against several diseases in the rhizosphere [151]. Many NPs themselves work as nanofertilizers or as a nanoencapsulating agent to transform conventional fertilizer into nanofertilizers [152]. The combination of biofertilizer (*Piriformospora indica*, a plant-growth-promoting fungus) and copper nanoparticles (Cu NPs) on *Cajanus cajan*, a leguminous crop, demonstrated that this combination application of nano + biofertilizer (nanobiofertilizer) might stimulate plant growth and vitality more effectively [153]. The list of nano-enabled agricultural products which are approved and manufactured for import in different countries is summarized in Table 3. Based on the research, nanofertilizers can improve the bioavailability of nutrients by increasing the shelf life of bioactive compounds in soil, thus, having a more obvious impact on crop growth [154].

### 3.3. Nanomaterials as Biotic and Abiotic Stress Alleviators

Plants are inevitably subjected to biotic and abiotic stress, which inhibits their growth and reduces yield, augmenting the global food crisis. NMs are regarded as effective and promising tools for overcoming the constraints in sustainable agricultural production by improving plant tolerance mechanisms under these stresses. Hojjat et al. stated in their investigation that biogenic Ag NPs improved the growth, length and weight of lentils and improved germination in *Trigonella foenum* during drought [155]. Various investigations reported that NMs restore plant development via increasing antioxidant activity, regulating metabolism and enhancing the quantity of photosynthetic pigments in plant systems subjected to plant stress [156]. It has been shown that plants can recover from oxidative stress after being treated with TiO_2_ NPs. TiO_2_ NPs improved the activity of antioxidant enzymes in corn tissues and additionally improved physiological functions and chlorophyll concentration by successfully minimizing Cd toxicity to the *Glycine max* plant [157]. Hence, the utilization of TiO_2_ NPs offers excellent application potential in reducing heavy-metal-induced plant oxidative stress. CeO_2_ NMs improved the salt tolerance of maize by maintaining Na^+^/K^+^ homeostasis, enhancing photosynthetic efficiency and reducing levels of ROS in salt-stressed *Zea mays* leaves [158]. The application of silicon dioxide improved plant growth parameters by lowering levels of hydrogen peroxide, electrolyte leakage and malondialdehyde. Furthermore, it decreased chlorophyll degradation while increasing stomatal conductance, net photosynthetic rate, transpiration rate and water use efficiency [159]. In a study by Rezvani et al., 10-day floodingstress reduced the root biomass, number of roots, leaf biomass and root length in the medicinal and aromatic plant species *Crocus sativus* [160]. Soaking the *C. sativus* corms in 40 or 80 ppm concentrations of nanosilver mitigated the negative effects of flooding stress and increased root development. When Al_2_O_3_ NPs of 30 to 60 nm were examined on the *Glycine max* under flooding conditions, their root length increased while glycolysis-related mitochondrial proteins expression repressed [161]. Iqbal et al. conducted experiments to find the effect of Ag NPs on wheat growth under heat stress. Ag NPs synthesized using a plant extract from *Moringa oleifera* were sprayed on *Triticum aestivum* at the three-leaf stage in various quantities. Exposure to heat stress alone decreased the dry biomass, but *T. aestivum* treated with Ag NPs at concentrations of 50 and 75 mg/L was found to be protected from heat stress and showed significant improvement in growth [122]. Petal longevity in *Pelargonium zonale* was increased after being treated with Ag NPs, which have been proven to mitigate the negative effects of dark-stress-induced oxidative damage [162]. They also examined reduced petal abscission in geranium cultivars subjected to nano silver and thidiazuron under dark preservation. NMs exhibit antibacterial properties against nematodes, bacteria and fungi that cause plant diseases in addition to abiotic stress. For instance, a ZnO nanoparticle made from a flower extract showed antibacterial action against *R. solanacearum* and decreased tomato bacterial wilt illness [163]. In contrast, a ZnO NPs made from *Citrus medica* peel extracts showed antimicrobial activity against *Bacillus subtilis*, *Streptomyces sannanesis*, *Salmonella enterica* and *Pseudomonas aeruginosa* [15]. According to Boxi et al., two prominent phytopathogens, *Fusarium solani* (which causes Fusarium wilt illnesses in potato and tomato plants) and *Venturia inaequalis* (which causes apple scab disease) were both inhibited by TiO_2_ nanoparticles at 0.75 and 0.43 mg/plate [164]. A TiO_2_ nanoparticle foliar spray in poinsettia and geranium (25 and 75 mM), as well as cucumber (1.6%) and poinsettia and geranium (1.6%), showed antibacterial action against pathogens *Pseudomonas cubensis* and *Pseudomonas syringae* pv. *lachrymans* and *Xanthomonas hortorum* [165,166]. All these experimental results validate the significant role of NMs in mitigating both biotic and abiotic stress and thereby enhancing plant growth and yield through numerous plant physiological mechanisms. It is clearly noticeable that NMs reduce the impact of environmental stresses on plants and help to improve the crop yields.

### 3.4. Biodegradable Nanoencapsulated System and Its Application in Agriculture

In order to enhance agricultural productivity, the use of agrochemicals has become increasingly prevalent. Among the many nanotechnological processes applied in the agricultural industry, micro-/macro- and nanoencapsulation are particularly noteworthy. These techniques involve the entrapment of bioactive compounds which can then be released in a controlled manner under specific conditions. This approach offers enormous potential for the development of agrochemicals with targeted chemical compositions and enhanced efficiency. Additionally, the agricultural sector benefits from a wide range of nano-based products, such as nanofertilizers, nanopesticides, nanofungicides, food and nutraceuticals, which are being used to promote sustainable farming practices, improve crop yields and fortify food supplies. By utilizing nanoencapsulation techniques, fertilizers can be modified to improve their efficiency and ensure a delayed release of entrapped bioactive compounds.

Encapsulation addresses the challenges of agronomical practices and minimizes the agrochemical load in practices [167]. One of the key benefits of encapsulating active ingredients is the gradual release of these substances to the crop. The sustained release intensifies the usage of resources in the best possible way, along with enhancing overall environmental safety [168]. Yet, it is worth noting that the primary focus of encapsulating active compounds has predominantly been on pesticides [169] and fertilizers [170]. In a study, it was demonstrated that chitosan is a great carrier for essential plant microbes [171]. *Azospirillum brasiliense* and *Pseudomonas fluorescens* were encapsulated in a chitosan–starch formulation, and this was applied to generate a controlled-release fertilizer [172]. The starch was added as filler to a chitosan-based formulation and employed as the crosslinking agent. The synthesized bacteria maintained a high vitality (109 colony-forming units (CFUs) of *A. brasilense*/g and 108 CFUs of *P. fluorescens*/g) for at least 12 months at room temperature and humidity. With their application to soil, bacteria gradually multiplied over the first 20 days before declining. Because of encapsulation, many targeted risks can be tackled [172]. 

Due to their adaptability, compatibility, nontoxicity and permeability, polymers have long served as encapsulants [173]. For the specific, controlled release of micromolecules, natural molecules such as chitosan, alginate, cellulose, starch, modified polysaccharides, carboxymethyl cellulose (CMC), Xanthan gum and gum Arabic have been employed as encapsulators. According to Tesfay et al., covering avocado fruits with 1% CMC and moringa leaf extract dramatically enhanced fruit quality and slowed the process of ripening [173]. The biological synthesis of ZnO NPs was performed by Saekow et al., who also evaluated the impact of ZnONP-loaded CMC on tomato quality characteristics and the capability of this mixture to combat Alternaria alternatives [174]. Some artificial polymers such as polyvinyl alcohol (PVA), polystyrene and polyalkylene glycol (PAG) have also been linked to similar uses. These polymers contain growth regulators such as metals, amino acids and other macro- and micronutrients, as well as pesticides, insecticides and herbicides [138].

Several studies have been performed to validate the properties of encapsulating an active ingredient with polymers. Alginate is employed in the agricultural sector as an absorbent polymer for the purpose of seed and fruit coating, a transporter of microbes and an enhancer of microbial bacterial activity for promoting plant growth. Agrochemical formulations use alginate encapsulation particularly to regulate the release of active chemicals [175] and the release of pesticides [176,177] or fungicides. Improved growth conditions for wheat plants were observed in stress conditions when encapsulated formulations of *Burkholderia cepacia* and *Pseudomonas fluorescens* were administered [178].

Chitosan, a potent natural polymer, has earned an illustrious presence due to its extraordinary qualities in terms of biocompatibility, chemical resistance and biodegradability. It is a popular eco-friendly substitute in the field of agriculture which is now widely utilized in a variety of agricultural techniques, including in biopesticides, seed treatment agents, biofertilizers, soil conditioners and growth enhancers [179]. A study showed new soil conditioner systems that address both soil fertilization and water-holding capacity. These systems were created via in situ hydrogelation of chitosan with salicylaldehyde in the presence of urea fertilizer. Chitosan has additionally been used to co-encapsulate resveratrol and curcumin [180]. Nanocomposite films from chitosan can effectively control infections by *Penicillium chrysogenum* and *Aspergillus* species by preventing their proliferation [181].

**Figure 2:**
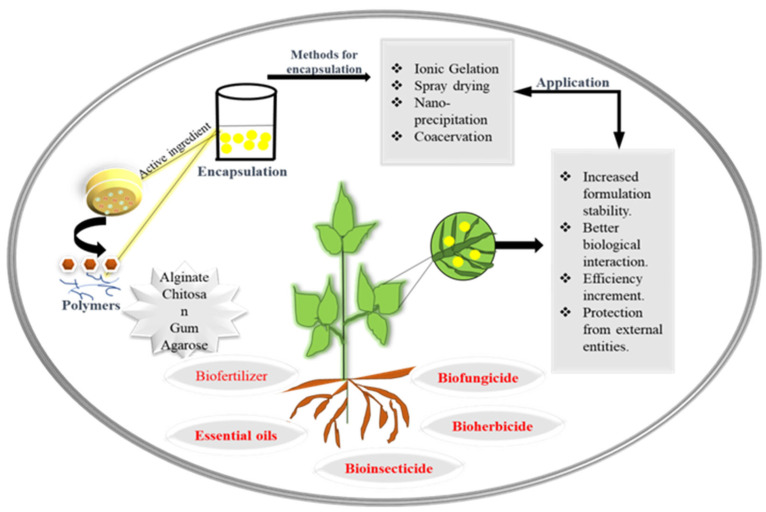
Systematic representation of encapsulation of bioactive compounds and their agricultural applications.

There are many types of red macroalgae (Rhodophyta) that contain the hydrophilic polysaccharides known as carrageenans (CGs). These are absorbent polymers that can help the soil retain water, offering a crucial support for plants during droughts [182]. Arabic gum is also used in agriculture due to its distinct structure, which includes a low viscosity and a high solid content, which makes it a good wall material for bacterial encapsulation. In modern encapsulation technology, particularly in the spray-drying method [183], Arabic gum is combined with other polymers, such as polysaccharides. A formulation for reducing tick harm was created in 2018 by Oliveira and her coworkers. This formulation, which contains *Pseudomonas* spp., Arabic gum, chitosan polymer and sodium casein, was created using the encapsulation process and applied to tomato and pepper plants to control adult mites [184] (Figure 2). Another study employed *P. putida* and their delivery system made up with chitosan, Arabic gum and sodium alginate against *Helicoverpa armigera* (Lepidoptera: Noctuidae), one of the most significant pests of cotton, for counting and manufacturing [185].

## 4. Uptake and Presence of Nanoparticles in Plants

In a soil system, the bioavailability, transport, fate and toxicity of nanoparticles are governed by soil factors such as organic matter, soil type, pH and moisture contents because these factors induce a series of changes in NP chemistry such as agglomeration, aggregation, dissolution and biotransformation [186,187]. Nanoparticles adsorbed firstly by the root system can be translocated to the aerial portion where they start accumulating in cellular or subcellular organelles [188]. The adsorption of nanoparticles by plant root surfaces is the first step of bioaccumulation. Studies suggest that silver nanoparticle uptake in the root is also strengthened by the acidic ambiance of the root cap [189] and iron plaque in the plant root [190].

The right size of NP facilitates their entry via biological pores (cell walls and stomata) revealing size as the crucial determinant for adsorption in plants [191,192]. A study proved that the adsorption of 50 nm copper NPs in wheat roots caused changes in root cell morphology and observed the existence of Cu NPs adhering to the root surface through SEM-EDS analysis [193]. NP agglomeration and reactivity with plant cell surfaces are correlated with the shape of NPs [194]. The surface charge and hydrophobicity of plant cell surfaces also act as critical determinants of the NP uptake process. Barrios et al., in their study, examined the uptake behavior of Ce NPs (coated and noncoated or bare Ce NPs). They suggested that surface coating reduces the uptake efficiency of NPs, but their translocations in the aerial portion remain unaffected [195]. The above-described facts support the design of NP ecotoxicological-based studies to understand the exact toxic mechanisms of NPs in plant systems. It is reported that the root epidermal cell allows the passage of small-sized NPs (3–5 nm) either directly through the biological pores or along with capillary and osmotic force. After crossing the root cell wall, NPs follow two basic pathways in the root epidermis to reach the vascular system. The apoplastic pathway is the most studied pathway for NPs transport, where NPs diffuse into the intercellular space of the cell wall and plasma membrane (without crossing the cell membrane) until they reach the vascular system, allowing the xylem to transport NPs unidirectionally upward [196,197]. A previous study reported that lanthanum oxide (La_2_O_3_) NPs increase the essential component of apoplastic barrier lignin by 1.5-fold and drastically reduce stomatal conductance and transpiration rate, subsequently causing significant growth inhibition [198]. Recently, another study also confirmed that Ce NPs promote endodermal suberization in the root of the *Sedum alfredii* plant [197]. NPs can cross the casparian strip symplastically to enter the vascular cylinder. Here, this process is promoted by the endodermal cell membrane’s carrier proteins through endocytosis or via pore formation. NPs move through the xylem into the aerial portion and through the phloem back to the roots [188].

Uptake of Ag NPs may also occur through the leaf, and the large pore size of leaf stomata facilitates entry of Ag NPs [199]. The uptake of Ag NPs through leaves in *Salvia officinalis* suggests that it can pass the cell wall and plasma membrane and enter the cell through endocytosis. It is localized in the cytoplasm and intercellular space of *S. officinalis* leaves [200]. After entering the body, NPs are distantly transported along with the sugar flow of the phloem sieve tube. Transportation of NPs by phloem allows bidirectional movement and accumulation in different parts of the plant. It is widely agreed that the apoplastic route favors the passage of water nutrients and nonessential metal complexes [201]. Translocation factors (TF) are the ratio of NP levels in the shoot and root. TF vary with particle properties and plant species. The small-size nano copper nanoparticles (nCu NPs) tended to transfer upward more feasibly than Cu NPs in *Cucumis sativus* that was exposed to 100 mg/kg Cu for 65 days [202]. Toxicity and bioaccumulation of NPs are influenced by total surface area and size; likewise, in *Lolium multiflorum*, Ag NPs (6 nm) were more toxic than (25 nm) Ag NPs because of their higher accumulation [203]. Foliar uptake of an NP is influenced by application methods and the shape, size, concentration and surface properties of the NPs. Leaf morphology (leaf area, size of stomata and cuticle thickness) also affects the trapping of NPs on the surface of the leaf [199,200]. The accumulation rate of NPs depends on the route of exposure. A study performed on *Glycine max* seedlings revealed more accumulation of silver in foliar exposure than in root application in leaves [204]. Recently, another study concluded that different forms of silver nanoparticles (20 nm Ag_2_S NPs, 3–8 nm Ag NPs, 50 nm Ag NPs and AgNO_3_) had differing uptake and bioaccumulation in the life cycle of *Brassica rapa*. They showed that pristine Ag NPs accumulated 14 times more Ag than other sulfide silver forms [205]. Therefore, a major factor determining the difference in uptake and NP accumulation is the stability of the NPs.

## 5. Nanomaterial Interaction with Plants 

**Figure 3:**
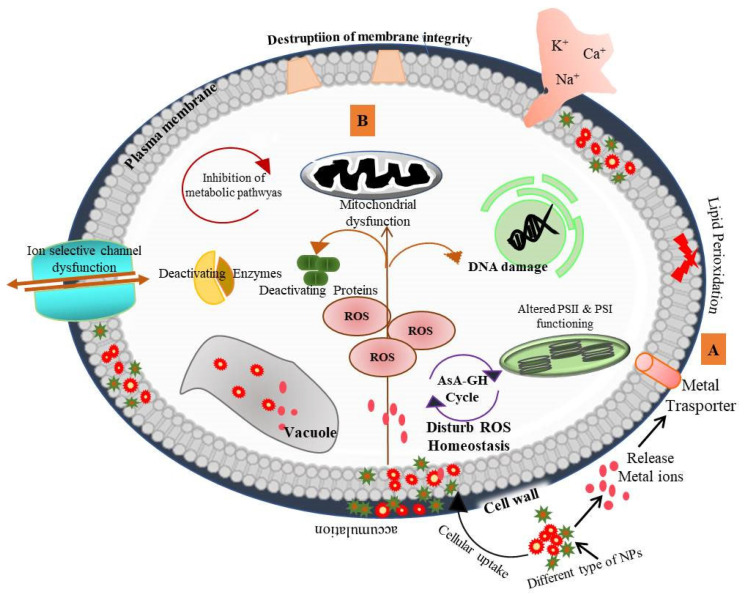
Cellular toxicity mechanism of various nanoparticles in plants. [A]—Direct attachment of NPs to the cell surface releases metal ions which enter through ion channels and accumulate in cell wall intracellular space and vacuoles. Uptake and accumulation of NPs varies with the characteristics of NPs (shape, size, surface and charge). [B]—NP stress causes reactive oxygen species (ROS) generation and induces damaging responses [217] available at CC BY 4.0.

The nanotechnology market has expanded significantly in agricultural sectors. Various nano-enabled agrochemical products have been launched in the market that include fertilizers for crop production, pesticides for disease resistance and nanosensors for monitoring plant health and soil quality. The emergence of nanotechnological applications in consumer products has also raised ethical and social concerns, including those about environmental safety issues. Plants, being the producers, are the most essential components of the terrestrial ecosystem. In the past few years, a large number of studies on the phytotoxic effect of NMs have been reported, but the mechanistic action of toxicity is still not well understood. Small-size NPs can easily be taken up by the plant and induce cytotoxic effects (Figure 3). The accumulation of NPs in different plant parts has been confirmed in *Oriza sativa* [206], *Lycopersicon esculentum* [207], *Cucumis sativus*, *Triticum aestivum* [194], *Sinapis alba* and *Lepidium sativum* [208]. The accumulation of NPs causes dysfunction of photosystem II (PSII) and PSI and ultimately affects photosynthetic rate [209]. For example, wheat chlorophyll content, photosynthetic rate and efficiency of PSII were suppressed by P25 (a commercial formulation of TiO_2_ NPs) [210]. The toxicity of cerium oxide NPs (25 nm) was also observed by measuring photosynthetic activity in *Glycine max*. CeO_2_ NPs-exposure-induced changes in the thylakoid membrane, which subsequently reduced chlorophyll content and inhibited the quantum yield of PSII [204]. Systemic biological approaches have been implemented to resolve the behavior of genes, proteins and metabolites in response to NMs interaction. In plants, gene expression analysis is the most studied predictive marker of NPs phytotoxicity through which NPs-induced phenotypic changes can be correlated. Likewise, a dose-dependent phytotoxic study of Cu NPs was performed in *Lactuca sativa*. Transcriptomic analysis suggested that the ATP-binding cassette (ABC) transporter or other metal-binding protein transporters are actively involved in Cu NPs accumulation, and an increase in the expression of antioxidants (POD, MDHAR, APX and FSDs) was also observed under NPs stress [211]. Recent analysis on Zn ONP- and MWCNT-exposed *Arabidopsis thaliana* seedlings suggested that inorganic NPs induce stronger inhibitory effects than MWCNT via analyzing the expression pattern of genes involved in ROS homeostasis [212]. NPs exposure in plants influences the expression of genes catalase (CAT), ascorbate (APX) [213], superoxide dismutase (SOD) [214], auxin signaling F-box protein and DNA mismatch repair protein (MSH5) [215]. In response to NPs stress, multiple metabolomic pathways are differentially regulated in order to protect the plant from oxidative damage. In this manner, the phenolics and amino acid synthesis are controlled in stress conditions via the phenylpropanoid pathway, glutathione metabolism, GABA shunt, shikimate pathway and flavonoid pathway [216,217]. Researchers are also trying to evaluate the behavior of NPs with other soil contaminants. A study reported the comparative toxic behavior of mixtures of NPs. The study showed that combined NPs binary systems have a lower toxic impact on seed germination and root growth than individual ones [218]. Toxicological-based studies showed the effect of various factors such as the shape, size, coating, composition and physiochemistry of the NPs on their phytotoxic behavior. In *S. lycopersicon*, CuO_2_ NPs accumulation was higher compared to in Al_2_O_3_ NPs and consequently caused mitochondrial membrane defects and changes in growth dynamics [143]. NPs also impugn the rhizospheric soil microbial community and functions. Ag NPs treatment negatively influenced the bacterial and fungal microbiota of the *Populus nigra* plant [219]. Direct application of CuO_2_ NPs as fertilizer inhibits important soil nitrification kinetics and diminishes the activity of soil nitrifiers [220]. Copper NPs are used as a component of fertilizers, but Cu NPs drastically affect microbial nitrogen cycle processes (nitrification and denitrification). They also decreased the activity of heterotrophic microbe populations in *Triticum aestivum* rhizospheres [221]. The rhizospheric microbial community plays a crucial role in maintaining plant growth and soil health. The non-target effect of NPs application should be monitored to sustain the biogeochemical process.

## 6. Soil Health and Biodiversity: Engineered Nanomaterials (ENMs) in Soil

It is not possible to gauge the concentration of NMs pollution in the environment due to a lack of proper detection tools and analytical approaches, so modeling predicted environmental concentrations are used. Based on the NMs production volume, it is estimated by a prediction model that the highest concentrations are expected for carbon-based NMs, followed by titanium oxide NPs and copper NPs in the aquatic systems; however, in soil, the highest concentrations are assumed for CeO_2_ and TiO_2_, followed by other NMs [222]. This information infers that NMs pollution reaches the soil by the end of the shelf life of nano-enabled products. ENMs enter the soil either by direct application or indirectly via atmospheric deposition, sludge application or agricultural irrigation [223]. The burden of NM pollution is much higher in soil than in water and air due to their low mobility [224]. Therefore, soil becomes the final sink for NMs released into the environment. Once incorporated in the soil matrix, NMs threaten the stability and function of the soil ecosystem. NMs interact with the soil’s organic and inorganic components and undergo a series of environmental transformations. Furthermore, NMs alter the porosity of soil, influencing the water dynamics and soil aggregation properties. Uncontrolled release of NMs into the soil matrix can have an adverse effect as they may potentially aggregate and not undergo degradation in the soil. Studies by Cao et al. and Kolesnikov et al. reported the impact of NMs on soil microscopic properties where ENMs at high concentrations negatively affected the dehydrogenase enzyme activity [225,226]. The effect of nanoparticles on soil enzymatic activity is varied by the nature of the nanoparticles; for example, the degree of influence on enzymatic activity in a soil sample is not the same when exposed to different NPs (Cu-, Ni- and Zn NPs). Catalase activity is greatly affected by the presence of Zn NPs in comparison to Ni- and Cu NPs. The overall enzyme activity of soil is sensitive to metal NPs in an order of Cu = Zn > Ni [226]. The duration of the NPs exposure also has a significant impact on enzymatic activity. As observed in silver-treated soil, the activities of -glucosaminidase, glucosidase, phosphatase and arylsulfatase decreased after 1 h and 1 week of treatment [227]. Another of the most studied issues of NM application in soil is their negative effect on soil microbial communities and the soil nutrient cycle. A study by Chen et al. showed the effect of multiwalled carbon nanotubes (MCNT) on soil enzyme activity and the diversity of rhizospheric microbial communities. They demonstrated the increased urease, phosphatase and sucrose activities under MCNT; however, the availability of available nitrogen and potassium was negatively affected by MCNT. The soil microbial taxonomic compositions were changed by the influence of MCNT [228]. Some researchers attribute the significant shift in the composition of the soil microbial community to metal NPs, especially highly dissolved heavy metal NPs. By exploring the relationship between bacteria and nitrogen functional genes, it was found that CuO NPs positively influence N fixation in the bacterial community by significantly increasing the expression of *nifH* and *amoA* genes (involved in nitrogen fixation) and negatively influencing the genes *norB* and *nosZ* (involved in denitrification) in bacteria [229]. Table 4 shows that soil microbial functional diversity and abundance significantly change when exposed to engineered nanomaterial. The homeostasis of microbes in soil is critical for maintaining plant and soil health. In the soil matrix, NMs can also affect the behavior of other soil pollutants. Recently, many researchers have studied the interaction of NMs with other pollutants. According to available literature, elevated CO_2_ (eCO_2_) can mitigate the toxicity of nano-Cr_2_O_3_ with respect to microbial biomass, soil enzymatic activities and bacterial alpha diversity in loamy soil [230].

## 7. Environmental Concern of NMs (Toxicological Aspects)

The use of NMs in agroscience is expanding as a result of the population’s ever-growing demand for agricultural yields and more efficient methods to compensate for agricultural practices that are negatively impacting the environment [227]. In reality, the growth of precision farming and sustainable agriculture may be significantly impacted by nanotechnology. This strategy seeks to balance lowering inputs (such as fertilizers, pesticides and herbicides) with increasing agriculture output (i.e., crop yields). It monitors environmental variables and takes targeted action to achieve desired results [167,168]. Researchers are contemplating the potential negative impacts on human and environmental health due to emerging applications of nanotechnology in agriculture and other sectors influencing the global economy [237]. In fact, the intentional introduction of NMs into agro processes may have unforeseen health effects [238]. According to this scenario, bioaccumulation in the environment and food chain is one exposure route that could lead to increased uptake of nanomaterial residues by humans and other environmental entities.

The NPs, also known as nanostructured materials, frequently enter the soil, water and air in the environment. Lead and tin NPs, among others, have been shown to be extremely stable, stiff and non-degradable. Furthermore, these NPs have toxicological effects when they infiltrate the tissues and organs of plants, people and animals [239]. Furthermore, the usage of silver nanoparticles (NPs) in consumer goods is widespread and harms the environment of the aquatic system by altering fish, algae, bacteria and other aquatic animals [240].

The hazardous significance of nano-agrochemicals is determined by their configuration, the mass and structure of the NPs. Nanocomposites with crystal structures are more dangerous than those with amorphous structures because size and adverse effects of NPs are related and diminish the size of NPs. It has also been discovered that large doses of nanofertilizers can change molecules in a variety of ways and disrupt plant feeding. Moreover, IAA and ABA levels in plant cells can be lowered by an overdose of Cu NPs. Parallel to this, the application of Fe_3_O_4_ NPs to second-generation maize crops resulted in severe physiological damage due to a greater buildup of Fe, despite the fact that the same dose (100 ppm) of NFs was observed to be beneficial for first-generation maize [241]. The knowledge of the “dose effect” on the organism’s level should not be the sole basis for the ecotoxicological risk assessment of nano-agrochemicals. It should include a study of the lethal process starting at the level of the cell and cell organelles [211].

Although NPs have been extensively utilized in sustainability applications, it is still unclear how long-term exposure to them can have negative effects on both human health and the environment. As a result, an integrated risk analysis based on the life cycle of NMs is required, as well as an assessment of exposure and hazards using a predetermined method for testing and monitoring. Moreover, fewer toxic NMs are carbon based (e.g., fullerene, CNTs and graphene). NPs can be employed as an alternative to reduce the toxicity of NPs [212].

## 8. Future Perspectives

Nano-enabled technology and nano-based carrier system applications in agricultural sectors can undoubtedly tackle the challenges of the food security issues of growing populations and climate change. Nanotechnological application has developed in every sector by leaps and bounds, but our understanding of nanomaterial-associated environmental challenges is at its nascent stage. The development of suitable analytical technology for the detection of transformed NMs in environmental matrices is needed. This might be possible to a certain extent by modifying and customizing the currently available advanced techniques. Additionally, there is an urgent need to gauge the bioavailable portion of NMs to assess their toxicity in terrestrial ecosystems. As the nanomaterial market is expanding every year, strict guidelines, testing and legislation must be enforced to regulate the production, handling and disposal of NMs.

Sustainable development is the key to balancing the challenges of growth with its opportunities. Thus, ecofriendly processes should be used in the synthesis, administration and dissemination of NMs. Nanotechnological interventions such as nanopesticides, nanoherbicides and nanofertilizers have increased the output of agriculture. The research on how nano-enabled strategies or products produce the intended outcome is still lacking. The impacts of NMs’ interactions with living systems are defined by their size, shape, charge and hydrophobicity. The sensitivity of each species to the NMs is quite different. The ecotoxicological behaviors of any ENM are governed by their characteristics (i.e., type, size, surface charge, coating and crystal chemistry) and exposure conditions (i.e., concentrations, duration and soil physicochemical properties). A thorough characterization of the NMs’ epitope is therefore requisite for concluding the toxicological effects. Furthermore, MNMs’ influence on soil physicochemical properties should be evaluated completely. The transformed forms of different nanofabricated products in soil and their degradation should be fully investigated in environmental matrices.

In the past few years, NMs with a wide range of physical and chemical properties, such as type, size, surface charge, coating and crystal chemistry, have been made, dumped into the environment and deposited there. Future nanotechnology design requires a functional understanding of NMs’ interactions with biological systems. Complete knowledge of each NM’s potential toxicological aspects is an essentially limitless task. According to the evidence that is currently available, various NMs frequently demonstrate diverse toxicity tendencies in many complex biological systems, as well as in the environment. To address the gaps in the issues of nanotechnology and environmental safety in the future, a clear mechanistic mechanism of NMs toxicity in complex systems should be clarified.

## Figures and Tables

**Table 2:** Summary of biologically synthesized NPs using plant extract and their characteristics.

Nanoparticle	Plant Extract	Size	Application	Ref.
Ag NPs	*Azadirachta indica*	34 nm	Antimicrobial activity against *E. coli* and *S. aureus*	[112]
	*Origanum vulgare*	2–25 nm	Bioreducing agent and antimicrobial activity	[113]
	*Morus alba*	80–150 nm	Antibacterial activity	[114]
Au-Ag NPs	*Nigella species*	3–37 nm	Antioxidant, cytotoxicity, catalytic activities	[115]
	*Tribulus terrestris*	~7 nm	Antibacterial activity against *Helicobacter pylori*, as well as cytotoxicity and catalytic activities	[116]
Se NPs	*Vitis vinifera*	3–18 nm	Fabrication purpose	[117]
	*Ocimum tenuiflorum*	15–20 nm	Pharmaceutical applications	[118]
TiO_2_ NPs	*Azadirachta indica*	25–87 nm	Antibacterial activity against *E. coli*, *B. subtilis*	[119]
	*Citrus reticulata*	50–150 nm	Reduced environmental impact	[120]
ZnO NPs	*Passiflora caerulea*	30–50 nm	Potent antibacterial	[121]
	*Rhamnus virgata*	~20 nm	Antimicrobial and antioxidant as well as cytoyoxic activity	[122]
	*Aloe socotrina*	15–50 nm	Used in the drug delivery approach	[123]

**Table 3:** Manufactured and approved nanotechnology-enabled nano agro products/inputs.

Commercial Name of Product	Nanomaterial Compositions	Manufacturer	Current Status and Legislation Compliance	Nanomaterial Application	Country of Origin
Nano-Ag Answer^®^	Billions of microorganisms, sea kelp and mineral electrolytes	Urth Agriculture, Monterey, CA, USA	Commercialized	Nanofertilizer	United States (US)
Ready to Use Spray	Biohumus in size range 100–700 nm	GreenEarth-NanoPlant, Fort Myers, FL, USA	Commercialized US patents (U.S. 15/290,257, U.S. 15/429,380)	Nanofertilizer	US
NanoPro^®^	Silicon dioxide and humic acid	Aqua Yield Operations, LLC., Sandy, UT, USA	Commercialized compliance with OSHA HCS (29CFR 1910.1200) and WHMIS 2015 Regulations	Crop protection	US
NanoCS™	Nitrogen, phosphorus, potassium (NPK) and zinc			Nanofertilizer	
NanoN+™	1% urea nitrogen			Nanofertilizer (N-delivery)	
NanoPhos^®^	NA			Controlled delivery of nutrient	
NanoK^®^					
NanoGro^®^					
NanoStress^®^					
NanoCalSi^®^					
NanoFe™					
NovaLand Nano	NA	Land Green & Technology, Taipei, Taiwan	Commercialized	Plant growth stimulators	Taiwan
NANOCU^®^	Nano copper, 10%, adjuvants and chelating materials, 90%	Bio Nano Tech, Giza, Egypt	Commercialized	Plant protection (fungicide and bactericide)	Egypt
Nano Ultra-Fertilizer	Organic matter, 5.5%; nitrogen, 10%; P_2_O_5_, 9%; K_2_O, 14%; P_2_O_5_, 8%; K_2_O, 14%; MgO, 3%	Sino Myain Tai Eco Technology Co., Ltd. (SMTET), Yangon, Myanmar	Commercialized	Nanofertilizer	Myanmar
Nano Calcium	CaCO_3_, 77.9%; MgCO_3_, 7.4%; SiO_2_, 7.47%; K, 0.2%; Na, 0.03%; P, 0.02%; Iron-7.4 ppm; Al_2_O_3_, 6.3 ppm; Sr, 804 ppm; sulfate, 278 ppm; Ba, 174 ppm; Mn, 172 ppm; Zn, 10 ppm	PAC International Network Co., Ltd., Koln, Germany	Commercialized	Nanofertilizer	Germany
PPC Nano	M protein, 19.6%; Na_2_O, 0.3%; K_2_O, 2.1%; (NH_4_)_2_SO_4_, 1.7%; diluent, 76%	WAI International Development Co., Ltd., Singapore/Malaysia	Commercialized	Nanofertilizer	Malaysia
Nano Green fertilizer	Extracts of grain, soybeans, potatoes, corn, coconut and palm	Nano Green Sciences, Inc., Gwalior, India			India
Nano Urea	4% N as encapsulated nitrogen (20–50 nm)	Indian Farmers Fertilizer Cooperative Limited (IFFCO), Mumbai, India	Commercialized	Nanofertilizer	India
Tropical nano PHOS	Nano phosphorus	Geetharam Agencies, Sole Proprietorship (Individual), Kerala, India	Commercialized	Controlled delivery of nutrient	India
Geolife Nano Combi	16.6%Zinc + 3.8% magnese +3.8% copper	Geolife Agritech India Pvt. Ltd., Mumbai, India	Commercialized	Controlled delivery of nutrient	India
Magic Root 4th Generation Nano Plant Growth Promoter	Mastermix of plant hormones	Infinite Biotech, Ahmedabad, India	Commercialized	Plant growth stimulators	India

Note: Given table was produced with information which is available online through the companies’ websites.

**Table 4:** Effect of different ENMs on soil microbial activity.

Type of Nanomaterial	Concentration Range in Soil	Toxic Effects	Ref.
ZnO, TiO_2_ and CeO_2_	1000 mg kg^−1^	NPs hampered thermogenic metabolism and reduced soil Azotobacter, P-solubilizing and K-solubilizing bacteria and enzymatic activities	[231]
TiO_2_ NPs	5–100 mg kg^−1^ soil	TiO_2_ NPs at 100 mg/kg of soil reduced the biomass of total phospholipid fatty acid (PLFA) and microbial load in soil	[232]
Silver (Ag), zinc oxide (ZnO) and fullerene (C_60_)	100 mg kg^−1^ dry weight	Nitrogen transformation suppressed by all three NPs via inhibiting *Flavobacterium* and *Nitrospira*	[233]
La_2_O_3_, Nd_2_O_3_ and Gd_2_O_3_ nanoparticle	10, 50 and 100 mg kg^−1^	Altered the ammonia-oxidizing archaea (AOA) and ammonia-oxidizing bacterial (AOB) community	[198]
Polyvinylpyrrolidone-coated Ag NPs	1, 10 and 100 mg kg^−1^ soil	Ag NPs at 100 mg kg^−1^ reduced dominant *Nitrosospira* and *Nitrosomonas*, and *Nitrosovibrio* even disappeared	[234]
		Ag NPs reduced the dehydrogenase soil enzyme activity	
CuO NPs	27, 54, 123, 265 and 627 mg Cu kg^−1^ soil	Inhibitory effect over dehydrogenase and phosphatase enzyme activities	[235]
		No significant inhibitory effects on the soil microbial growth	
Ag NPs	1, 10 and 100 mg kg^−1^ dry soil	Reduced the urease enzyme and ammonia-oxidizing enzyme activities	[236]

## Data Availability

No data, models or code were generated or used during the study.
